# Effects of Shenmai injection against chronic heart failure: a meta-analysis and systematic review of preclinical and clinical studies

**DOI:** 10.3389/fphar.2023.1338975

**Published:** 2024-02-06

**Authors:** Yang Wu, Tianli Li, Pochen Li, HsuanChieh Peng, Ang Gao, Jisheng Wang, Haiyan Zhu, Xian Wang

**Affiliations:** ^1^ Department of Cardiology, Dongzhimen Hospital, Beijing University of Chinese Medicine, Beijing, China; ^2^ National Integrated Traditional and Western Medicine Center for Cardiovascular Disease, China-Japan Friendship Hospital, Beijing, China; ^3^ Department of Respiratory, Dongzhimen Hospital, Beijing University of Chinese Medicine, Beijing, China; ^4^ Medical Services Section, Dongzhimen Hospital, Beijing University of Chinese Medicine, Beijing, China; ^5^ Department of Geriatrics, Dongzhimen Hospital, Beijing University of Chinese Medicine, Beijing, China

**Keywords:** Shenmai injection, chronic heart failure, meta-analysis, preclinical studies, clinical studies

## Abstract

**Objective:** This study aims to evaluate the clinical and preclinical efficacy of SMI in treating CHF, and to summarize the relevant mechanisms of action in order to provide evidence for its role in CHF treatment.

**Methods:** A systematic computerized search of eight databases and three registry systems was performed, with the time frame spanning from the inception of the databases to 30 June 2023. Strict procedures were used for data extraction, quality assessment, and data analysis. The methodological quality of the included studies was assessed using RoB-2 and SYRCLE tools. Statistical analysis was performed using Rev Man 5.4 software, using either fixed-effects or random-effects models.

**Results:** A total of 25 clinical trials (including test group 1,367 patients, control group 1,338 patients) and 11 animal studies (including 201 animals) were included in this review. The meta-analysis of clinical studies showed that SMI can improve cardiac function indicators (LVEF, LVFS, LVEDV, LVESV, LVEDD, LVESD) (*p* < 0.00001), reduce BNP/NT-proBNP levels (*p* < 0.01), and improve inflammatory markers (hs-CRP, TNF-α, IL-6) (*p* < 0.00001) and endothelin (ET) levels (*p* < 0.0001). In animal studies, SMI demonstrated improved cardiac function (LVEF, LVFS) (*p* < 0.05), and improved heart failure markers (NT-proBNP, *p* < 0.05) when compared to control groups.

**Conclusion:** This study represents the first meta-analysis which includes both preclinical and clinical studies on SMI. Clinical and animal studies have shown that SMI can improve cardiac function in CHF patients through its anti-apoptotic effects, antioxidant activities, anti-inflammatory effects, and improvement of myocardial metabolism. This study has certain limitations in terms of literature quality, quantity, and follow-up time. Therefore, the conclusions drawn from this study may require further validation through larger-scale, high-quality RCT trials.

## 1 Introduction

Heart failure is a complex clinical syndrome characterized by abnormal changes in cardiac structure and/or function, resulting in impaired ventricular contraction and/or relaxation (McDonagh et al., 2023). With the aging of our population, the incidence rate of chronic heart failure (CHF) has been steadily increasing. In recent years, there have been some advances in the treatment of CHF (Heidenreich et al., 2022). In addition to the traditional use of diuretics, vasodilators, angiotensin-converting enzyme inhibitors (ACEIs)/angiotensin receptor blockers (ARBs), beta-blockers, and mineralocorticoid receptor antagonists, novel drugs such as sodium-glucose cotransporter-2 (SGLT-2) inhibitors have also been used clinically (Packer et al., 2020; Anker et al., 2021). However, the 5-year mortality rate of CHF remains high, and patients still experience a gradual decline in cardiac function, leading to poor quality of life (McDonagh et al., 2021).

From the perspective of traditional Chinese medicine (TCM), deficiency of both qi and yin is a common syndrome of CHF (Li et al., 2016a; Leung et al., 2023). Shenmai Injection (SMI), a Chinese patent medicine composed of Ginseng and Ophiopogon japonicus, is mainly used to tonify qi and nourish yin, and has been widely used in the treatment of CHF (Hu et al., 2005; Wang et al., 2010a; Ma et al., 2010; Huang et al., 2011; Zhang and Zhou, 2012; Li, 2013; Yin et al., 2013; Zhai, 2013; Li et al., 2016b; Cai et al., 2016; Guan et al., 2016; Liu, 2017; Luo, 2018; Meng et al., 2018; An and Xiao, 2019; Cui et al., 2019; Li, 2019; Qin, 2019; Shan and Qin, 2019; Ping and Zhao, 2021; Zhang et al., 2021; Wang et al., 2022; Wu and Li, 2022; Di et al., 2023; Xie et al., 2023). Several clinical studies have shown that the combine application of SMI with conventional Western treatment (CWT) has superior clinical efficacy compared to administering CWT alone in the treatment of CHF (Hu et al., 2005; Wang et al., 2010a; Ma et al., 2010; Huang et al., 2011; Zhang and Zhou, 2012; Li, 2013; Yin et al., 2013; Zhai, 2013; Li et al., 2016b; Cai et al., 2016; Guan et al., 2016; Liu, 2017; Luo, 2018; Meng et al., 2018; An and Xiao, 2019; Cui et al., 2019; Li, 2019; Qin, 2019; Shan and Qin, 2019; Ping and Zhao, 2021; Zhang et al., 2021; Wang et al., 2022; Wu and Li, 2022; Di et al., 2023; Xie et al., 2023). Animal studies suggest that SMI can increase cardiac output and improve cardiac function by ameliorating oxidative stress, improving myocardial metabolism, suppressing inflammatory responses, and improving endothelial function (Tan, 2005; Zhu et al., 2008; Zhang et al., 2009; Wang et al., 2010b; Wang et al., 2012; Xu, 2015; Wu, 2016; Cheng et al., 2021; Zhai et al., 2021; Li et al., 2023a; Hu et al., 2023). As the number of published literature increases, it has become necessary to comprehensively evaluate the clinical efficacy and safety of SMI as an adjunctive therapy for CHF by means of rigorous systematic reviews and meta-analyses.

**FIGURE 1:**
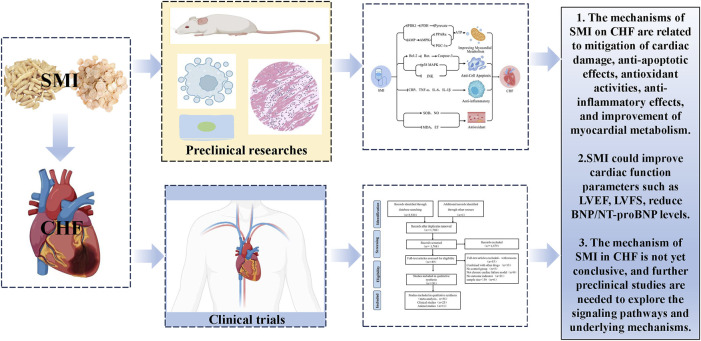
Graphical summary.

This study aims to investigate the therapeutic efficacy and mechanisms of SMI in CHF from both current preclinical and clinical perspectives. The primary focus of this research is to compare the clinical efficacy and safety of SMI as an adjunctive treatment for CHF compared to solely CWT, as well as to explore the key mechanisms by which SMI acts on CHF. The following is a graphical abstract of the article in Figure 1.

## 2 Data material and methodology

### 2.1 Search strategy and selection of databases

A computerized search was conducted in 8 databases (PubMed, The Cochrane Library, Web of Science, Embase, CNKI, WanFang Data, VIP, and SinoMed databases) and 3 registry systems (ClinicalTrials.gov, WHO International Clinical Trials Registry Platform, and the Chinese Clinical Trial Registry). The search aimed to retrieve randomized controlled trials (RCTs) and animal studies on the treatment of CHF with SMI. The search period ranged from the inception of the databases to 30 June 2023, using a combination of subject and free text terms. In addition, a manual search of relevant literature was performed. The search was restricted to articles published in English and Chinese. Detailed search strategies and results from the 8 databases are provided in the Supplementary Appendix.

### 2.2 Inclusion/exclusion criteria

#### 2.2.1 Inclusion criteria

1) Study type: Preclinical studies or RCTs.
2) Study population: Patients who were diagnosed with CHF in clinical studies; preclinical studies include models of pressure overload-induced heart failure or myocardial infarction-induced heart failure.
3) Intervention: In clinical trials, the intervention method for experimental groups is combined treatment with CWT and SMI, while control groups receive solely CWT or CWT plus placebo. In preclinical studies, the intervention measure in experimental groups is SMI administered at any dose, while control groups receive an equivalent amount of non-functional fluid (i.e., sodium chloride or distilled water) or altogether given no treatment.
4) Outcome indicators: Outcome indicators in clinical trials include: ① left ventricular ejection fraction (LVEF), ② brain natriuretic peptide (BNP) or N-terminal pro-brain natriuretic peptide (NT-proBNP), ③ overall response rate, ④ 6-min walk distance (6-MWD), ⑤ Cardiac function[left ventricular fractional shortening (LVFS), left ventricular end-diastolic volume (LVEDV), left ventricular end-systolic volume (LVESV), left ventricular end-diastolic diameter (LVEDD), left ventricular end-systolic diameter (LVESD)], ⑥ Mechanism indicators[high-sensitivity C-reactive protein (hs-CRP), tumor necrosis factor-alpha (TNF-α), interleukin-6 (IL-6), endothelin (ET)], ⑦ Adverse drug reactions. There are no specific requirements for outcome indicators in preclinical studies.

#### 2.2.2 Exclusion criteria

1) Studies including patients with acute heart failure (AHF), as well as CHF caused by chronic pulmonary heart disease or dilated cardiomyopathy.
2) Duplicate studies, reviews, clinical protocols, commentaries, case reports, etc.
3) Studies involving other Chinese herbs or related traditional Chinese medicine interventions other than SMI.
4) Studies for which research data cannot be obtained even after contacting the original authors.
5) Studies without a control group.
6) Studies with a sample size of less than 50 cases.

### 2.3 Data extraction

The following information was extracted from the final included literature by independent reviewers Yang Wu and Pochen Li: ① first author’s name and year of publication; ② specific information within the clinical studies, including age, sample size, and intervention measures, as well as species, number, and weight; ③ modeling and anesthesia methods for animal models; ④ outcome indicators, including clinical efficacy, mechanism indicators, adverse drug reactions, etc. If multiple observation time points were reported in the study, only results from the final time point were included.

### 2.4 Risk of bias in included studies

The quality of clinical trials was assessed using the RoB-2 tool. The RoB-2 tool includes domains such as randomization process, assignment to intervention, adherence to intervention, missing-outcome data, measurement of outcome, selection of reported outcome, and RoB-2 overall score (Sterne et al., 2019). Two reviewers (Yang Wu and Tianli, Li) independently assessed the quality of the studies. In case of disagreement, discussions or consultations with the corresponding authors (Xian Wang) were conducted to resolve the issues.

Animal studies were assessed using the SYRCLE Animal Risk of Bias tool. It includes 11 items, namely, sequence generation, baseline characteristics, allocation concealment, random housing, blinding of investigators, random outcome assessment, blinding of outcome assessors, incomplete data, selective outcome reporting, other sources of bias, and overall score (Hooijmans et al., 2014). Each item is assigned a score of “1”.

### 2.5 Statistical analysis

Statistical analysis was performed with Rev Man 5.4 software. The I^2^ test was used to assess heterogeneity. According to the Cochrane Heterogeneity Analysis Guidelines, I^2^ values in the range of 0%–40% may indicate low heterogeneity, while 30%–60% may indicate moderate heterogeneity, 50%–90% may indicate high heterogeneity, and 75%–100% may indicate substantial heterogeneity. If I^2^ ≤ 50%, a fixed-effects model was used; if I^2^ > 50%, a random-effects model was used (Higgins et al., 2019). A *p*-value less than 0.05 was considered statistically significant. Relative risk (RR) was used as the effect indicator for categorical data, mean difference (MD) was used as the effect indicator for continuous data, and standardized mean difference (SMD) was used when a particular outcome indicator was assessed using multiple measurement methods or different units of measurement.

### 2.6 Subgroup analysis

If there is substantial heterogeneity in the outcome, subgroup analyses can be conducted to assess the influence of variables and explore its source of origin.

### 2.7 Sensitivity analysis

Sensitivity analysis is performed on highly heterogeneous outcomes to indirectly identify sources of heterogeneity and to assess the stability and reliability of the combined results.

### 2.8 Publication bias

Publication bias of the main outcome is assessed using a funnel plot. The funnel plot is generated using Rev Man 5.4 software.

## 3 Results

### 3.1 Included literature

**FIGURE 2:**
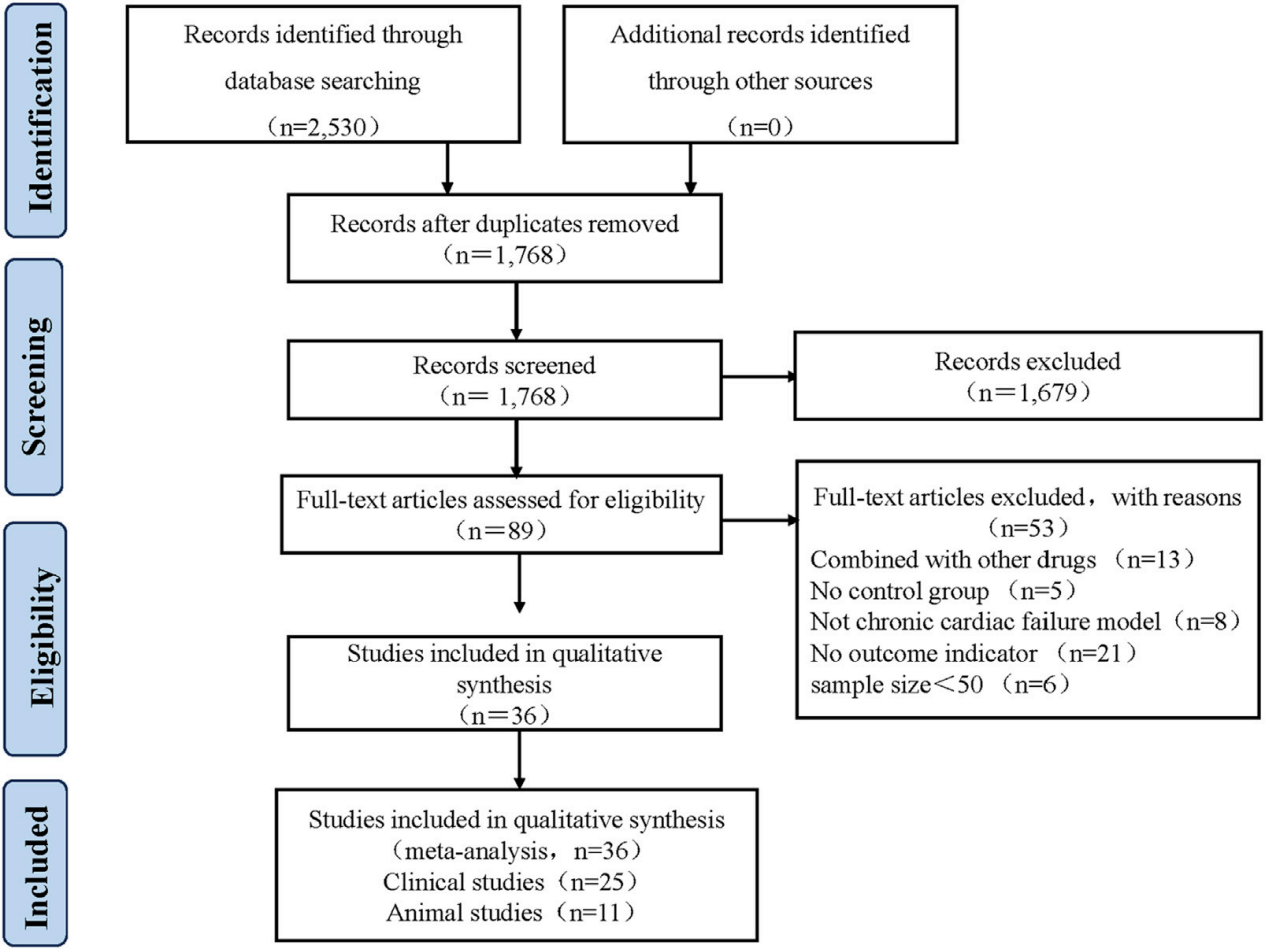
Literature screening process and results.

A total of 2,530 articles were identified from 8 databases. Repeat records were removed using Endnote X9 software, leaving 1,768 articles. Two reviewers Yang Wu and Pochen Li independently screened the titles and abstracts to select relevant articles, resulting in the selection of 89 articles. Both reviewers Yang Wu and Pochen Li thoroughly reviewed the full text of these 89 articles. In the end, 25 clinical studies and 11 animal studies published between 2005 and 2023 were included. Any discrepancies during the selection process were resolved by Xian, Wang. The flowchart of the selection process for the included studies is shown in Figure 2.

### 3.2 Characteristics of included studies

#### 3.2.1 Characteristics of clinical studies

The 25 included RCTs have sample sizes ranging from 64 to 198. The intervention in experimental groups was SMI combined with CWT, while control groups received CWT alone. The dosage of SMI ranged from 20 mL to 200 mL per day, and the duration of treatment ranged from 2 weeks to 12 weeks. Outcome indicators include LVEF, BNP or NT-proBNP, overall efficacy rate, 6-MWD, cardiac function indicators, mechanical indicators, and adverse drug reactions (Supplementary Table S1).

#### 3.2.2 Characteristics of animal studies

A total of 11 animal studies were included, of which 10 studies were published in Chinese journals and one published in an English journal. In terms of species, 8 studies used rats, while 3 studies used dogs. Regarding the sex of the animals, 8 studies involved male rats, while the sex of dogs was not specified in the remaining 3 studies. Among the studies mentioned, 4 studies specified anesthetic methods, including urethane, chloral hydrate, pentobarbital, and pentobarbital sodium. The other 7 studies did not specify anesthesia methods. Regarding modeling methods, 5 studies modeled myocardial infarction-induced heart failure and 6 studies modeled pressure overload-induced heart failure. All experimental groups received different doses of SMI as intervention, while control groups received sterile injection water, distilled water, 5% glucose, or 0.9% sodium chloride. The experimental periods ranged from 1 day to 4 weeks in all studies (Supplementary Table S2).

#### 3.2.3 Mechanisms of action in animal studies

Of the included animal studies, a total of 6 studies investigated the mechanisms of SMI in the treatment of CHF. The identified mechanisms mainly included anti-apoptotic effects, antioxidant effects, anti-inflammatory effects, and improvement of myocardial metabolism (Supplementary Table S3).

### 3.3 Bias risk in results

#### 3.3.1 Bias risk in clinical studies

All RCTs were considered to be at low risk for adherence to intervention and missing-outcome data. Some studies were considered to be at low risk with regard to the randomization process, assignment to intervention, measurement of outcome, and selection of reported outcomes, while others were considered to have some concerns (Supplementary Table S4).

#### 3.3.2 Bias risk in animal studies

11 animal studies were assessed using the SYRCLE bias risk tool. All studies had complete outcome data and no other biases were identified. Most of the animal studies had comparable baselines, and 4 studies had a low risk of random sequence generation. In some studies, the outcome evaluations were randomly selected (Supplementary Table S5).

### 3.4 Meta-analysis results of clinical studies

#### 3.4.1 LVEF

**FIGURE 3:**
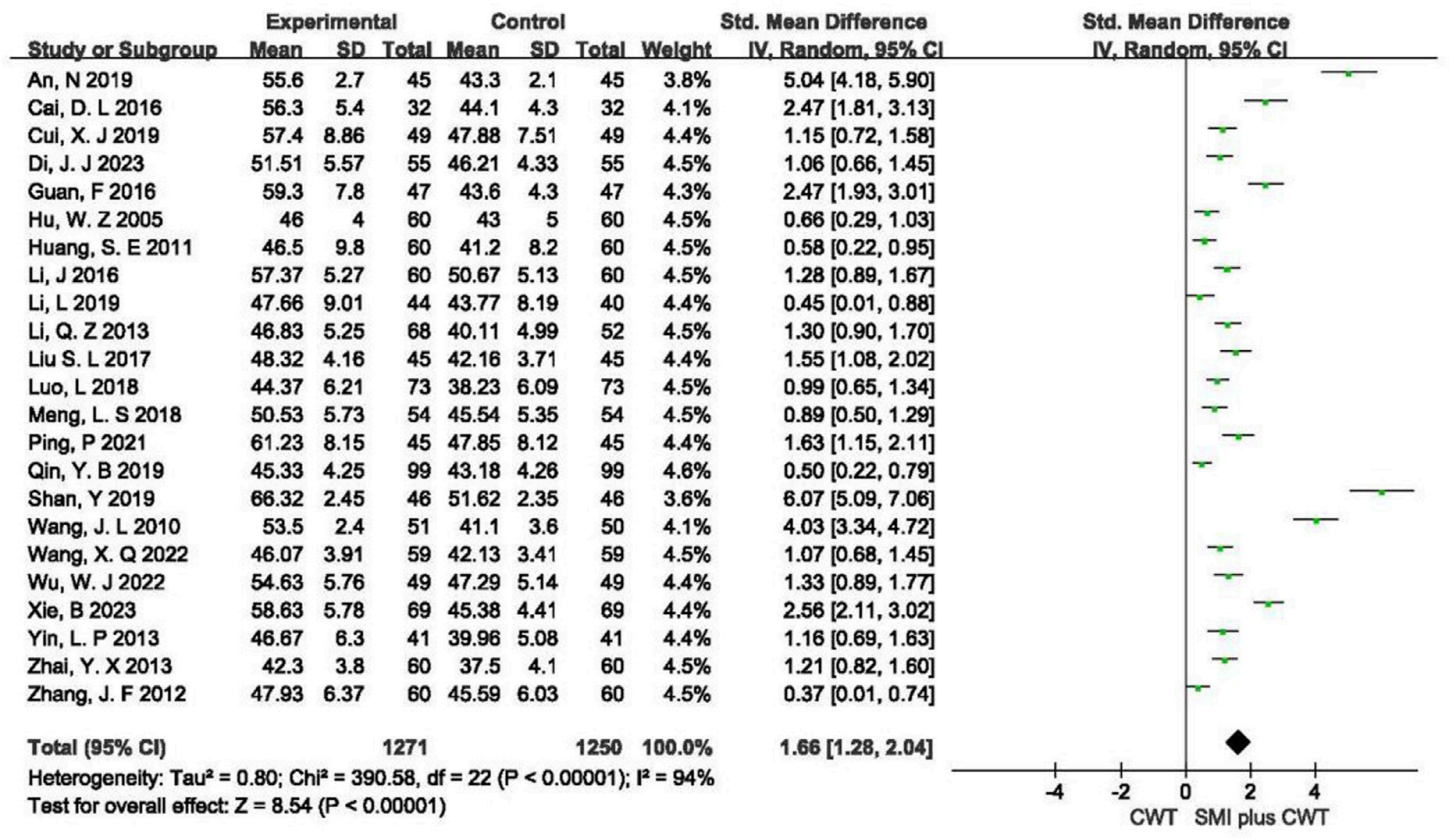
The meta-analysis result of the effect of LVEF.

A total of 23 RCTs with 2,521 patients were included. The results of the random-effects model meta-analysis showed that the concomitant of SMI during intervention signaled a significant improvement in LVEF compared with the control groups [SMD = 1.66, 95% CI (1.28, 2.04), *p* < 0.00001] (Figure 3).

#### 3.4.2 BNP/NT-proBNP

**FIGURE 4:**
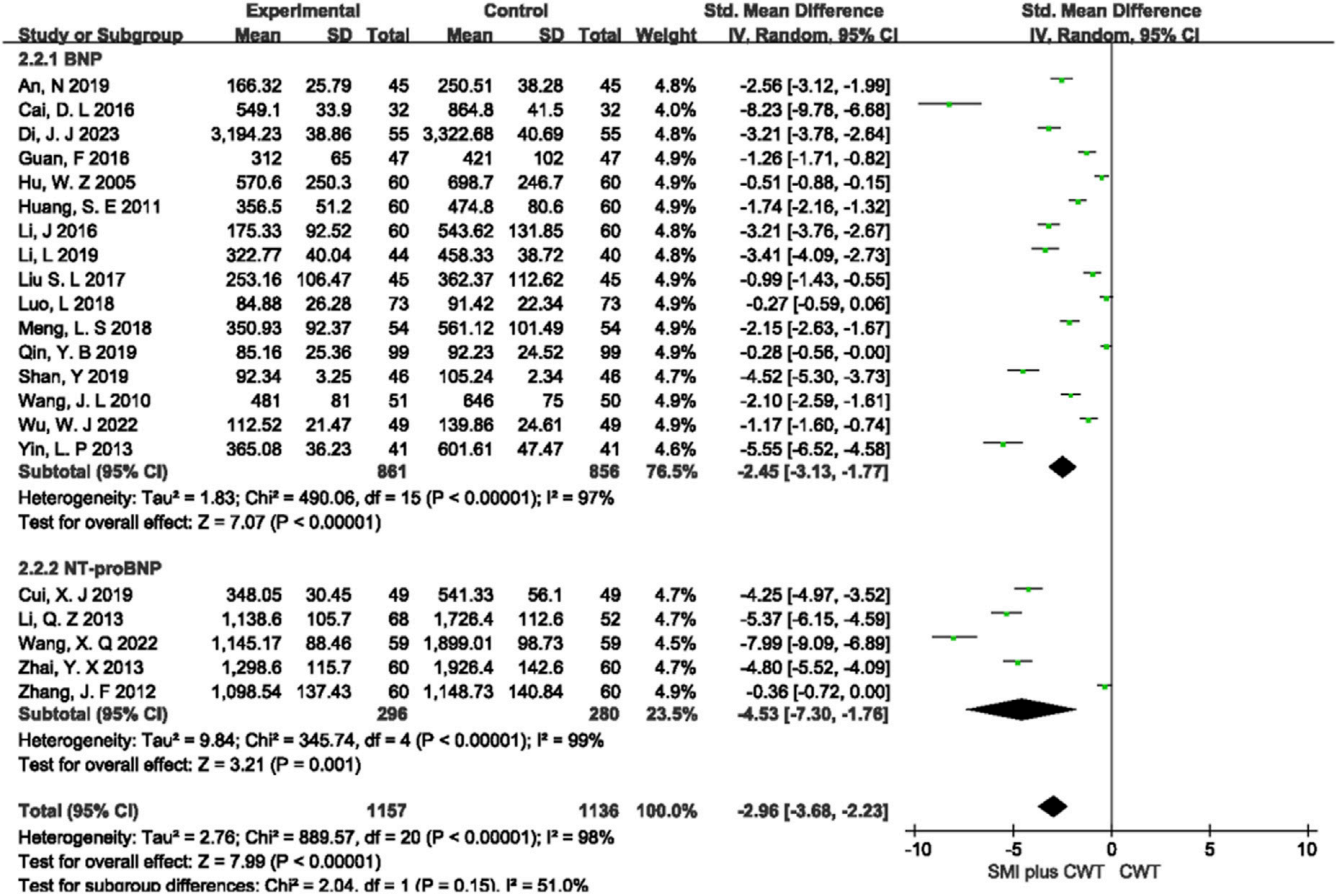
The meta-analysis result of BNP and NT-proBNP.

A total of 21 RCTs with 2,293 patients were included, of which 16 studies evaluated BNP and 5 studies evaluated NT-proBNP. The results of the random-effects model meta-analysis showed that the concomitant of SMI during intervention was associated with a significant reduction in BNP [SMD = −2.45, 95% CI (−3.13 −1.77), *p* < 0.00001] and a significant reduction in NT-proBNP [SMD = −4.53, 95% CI (−7.30 −1.76), *p* = 0.001] compared with the control groups (Figure 4).

#### 3.4.3 Overall response rate

**FIGURE 5:**
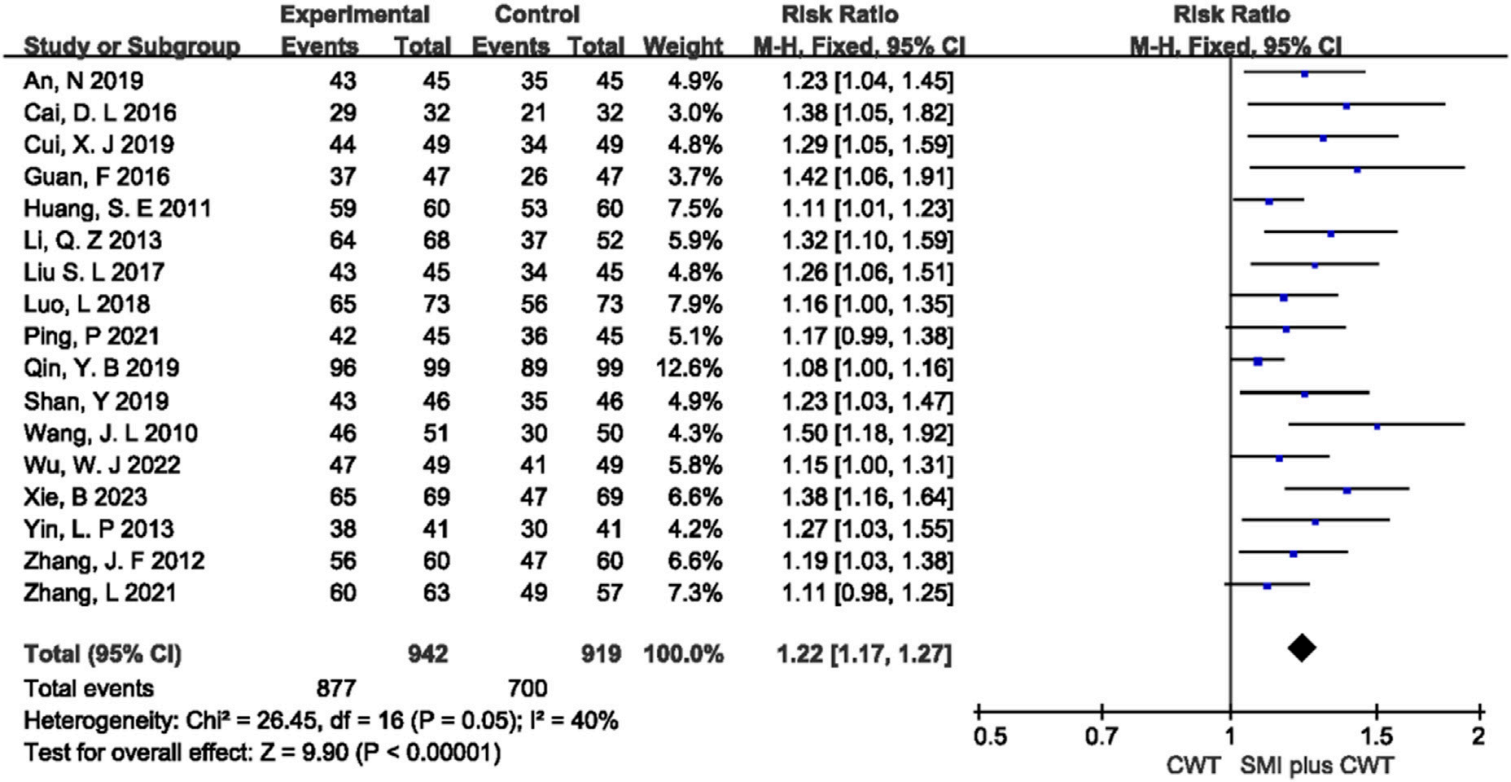
The meta-analysis result of total effectiveness rate.

A total of 17 RCTs with 1,861 patients were included. The results of the fixed-effects meta-analysis showed that the concomitant of SMI was associated with a higher overall response rate compared with the control groups [RR = 1.22, 95% CI (1.17, 1.27), *p* < 0.00001] (Figure 5).

#### 3.4.4 6-MWD

**FIGURE 6:**
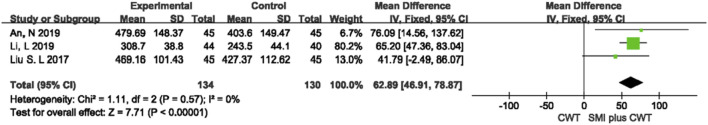
The meta-analysis result of 6-MWD.

Three trials with 264 patients were included. The results of the fixed-effects meta-analysis showed that the concomitant of SMI was associated with a greater 6-MWD compared with the control groups [MD = 62.89, 95% CI (46.91, 78.87), *p* < 0.00001] (Figure 6).

#### 3.4.5 Indicators of cardiac function

**FIGURE 7:**
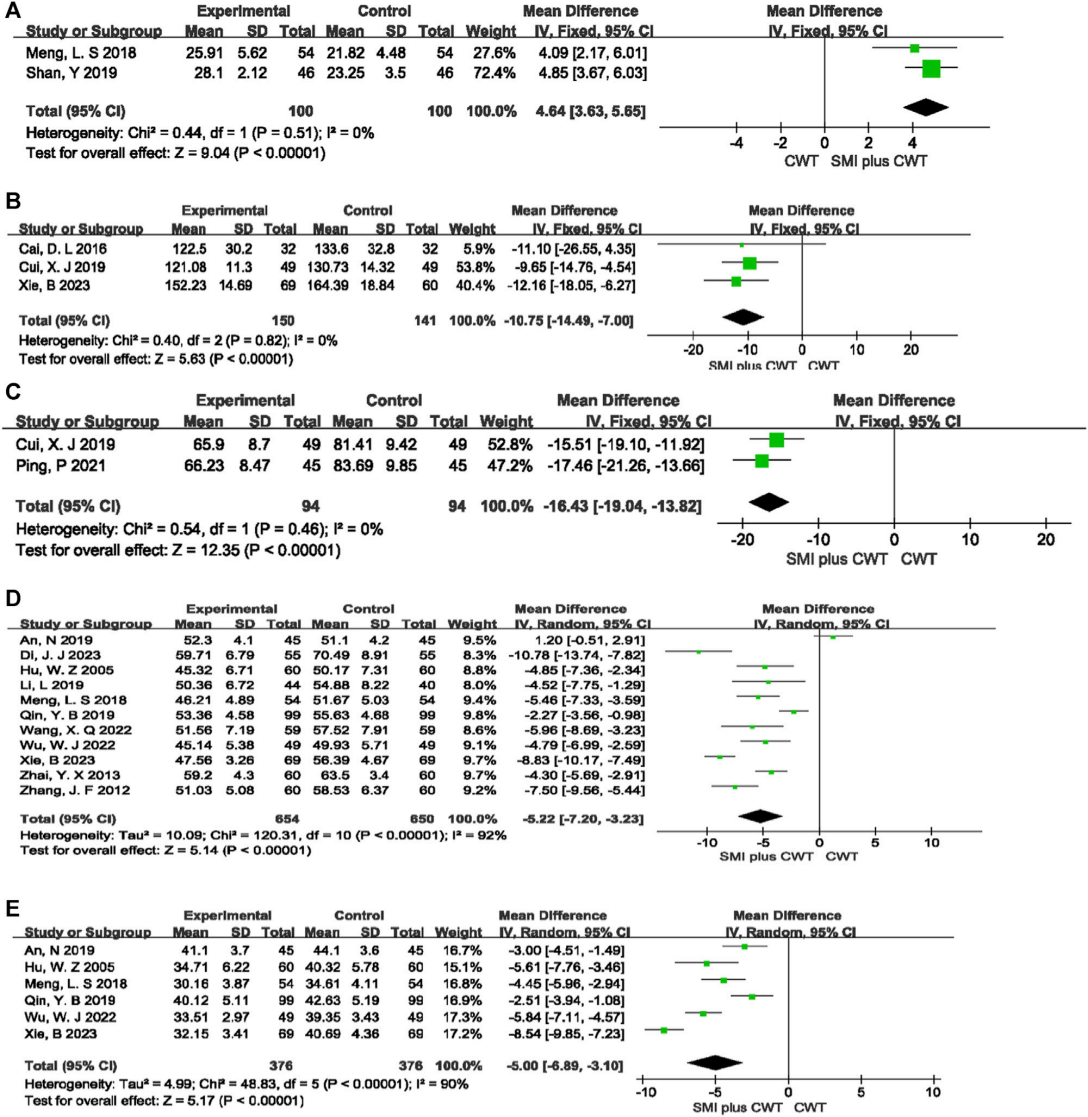
The meta-analysis result of indicators of cardiac function **(A)**:LVFS; **(B)**:LVEDV; **(C)**:LVESV; **(D)**:LVEDD; **(E)**:LVESD.

Two RCTs evaluated LVFS, 3 RCTs evaluated LVEDV, 2 RCTs evaluated LVESV, 11 RCTs evaluated LVEDD, and 6 RCTs evaluated LVESD. The results showed that, compared with the control groups, the concomitant of SMI during intervention was associated with a greater increase in LVFS [MD = 4.64, 95% CI (3.63, 5.65), *p* < 0.00001], a reduction in LVEDV [MD = −10.75, 95% CI (−14.49 −7.00), *p* < 0.00001], a reduction in LVESV [MD = −16.43, 95% CI (−19.04 −13.82), *p* < 0.00001], a reduction in LVEDD [MD = −5.22, 95% CI (−7.20 −3.23), *p* < 0.00001], and a reduction in LVESD [MD = −5.00, 95% CI (−6.89 −3.10), *p* < 0.00001] (Figure 7).

#### 3.4.6 Mechanistic indicators

**FIGURE 8:**
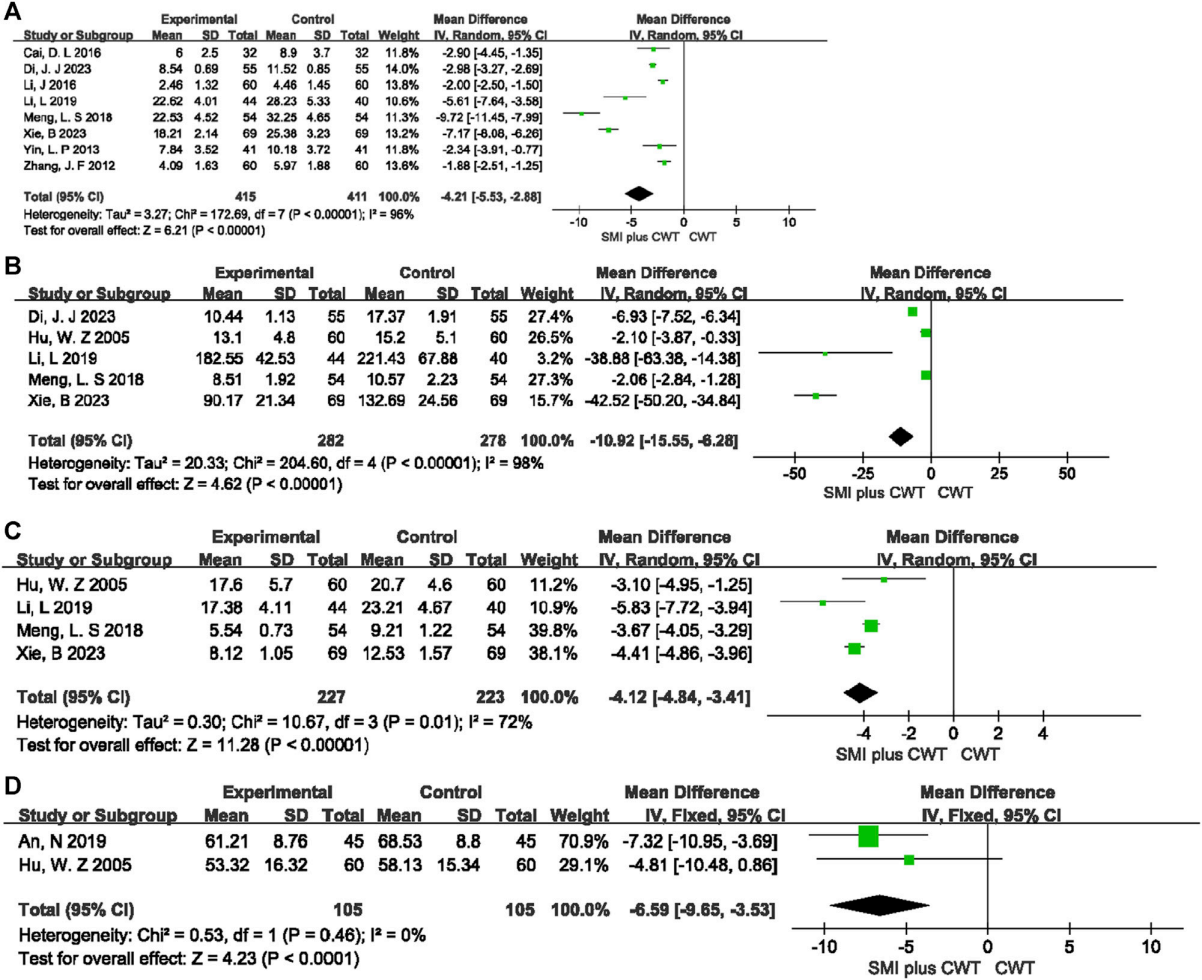
The meta-analysis result of mechanistic indicators **(A)**:hs-CRP; **(B)**:TNF- α; **(C)**:IL-6; **(D)**:ET.

A total of 8 RCTs evaluated hs-CRP, 5 RCTs evaluated TNF-α, 4 RCTs evaluated IL-6, and 2 RCTs evaluated ET. The results indicated that the concomitant of SMI during intervention was more effective in reducing hs-CRP [MD = −4.21, 95% CI (−5.53 −2.88), *p* < 0.00001], TNF-α [MD = −10.92, 95% CI (−15.55 −6.28), *p* < 0.00001], IL-6 [MD = −4.12, 95% CI (−4.84 −3.41), *p* < 0.00001], and ET [MD = −6.59, 95% CI (−9.65 −3.53), *p* < 0.0001] (Figure 8).

#### 3.4.7 Adverse drug reactions

**FIGURE 9:**
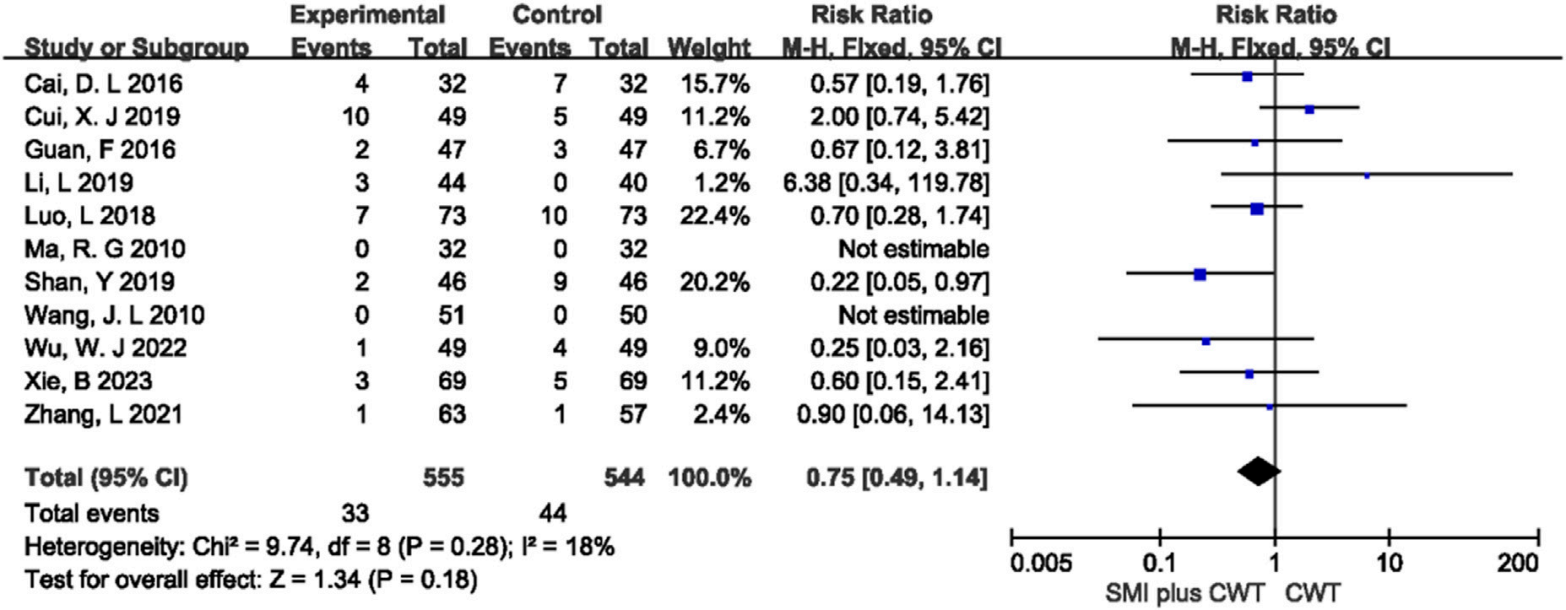
The meta-analysis result of adverse drug reactions.

A total of 11 studies reported the overall incidence of adverse events in the two groups. The difference between the concomitant of SMI group and the control group was not statistically significant (*p* = 0.18) (Figure 9). Among the 11 studies, 8 types of adverse reactions were reported, including gastrointestinal reactions, fatigue, dizziness and headache, rash or pruritus, abnormal liver and kidney function, hypotension, palpitations, and arthralgia. Meta-analysis was performed for 8 types of adverse reactions, and the results showed that there was no statistically significant difference (*p* > 0.05) in the incidence of these adverse reactions between the two groups (Supplementary Table S6).

### 3.5 Results of animal studies

Animal studies were divided into rat and dog studies, with 8 studies using rats and 3 studies using dogs. Meta-analyses were performed for outcome indicators of cardiac function (LVEF, LVFS), heart failure biomarkers (NT-proBNP), and mechanistic indicators (ET, TNF-α) in the 8 rat studies.

#### 3.5.1 Rat efficacy indicators

##### 3.5.1.1 Cardiac function indicators

**FIGURE 10:**
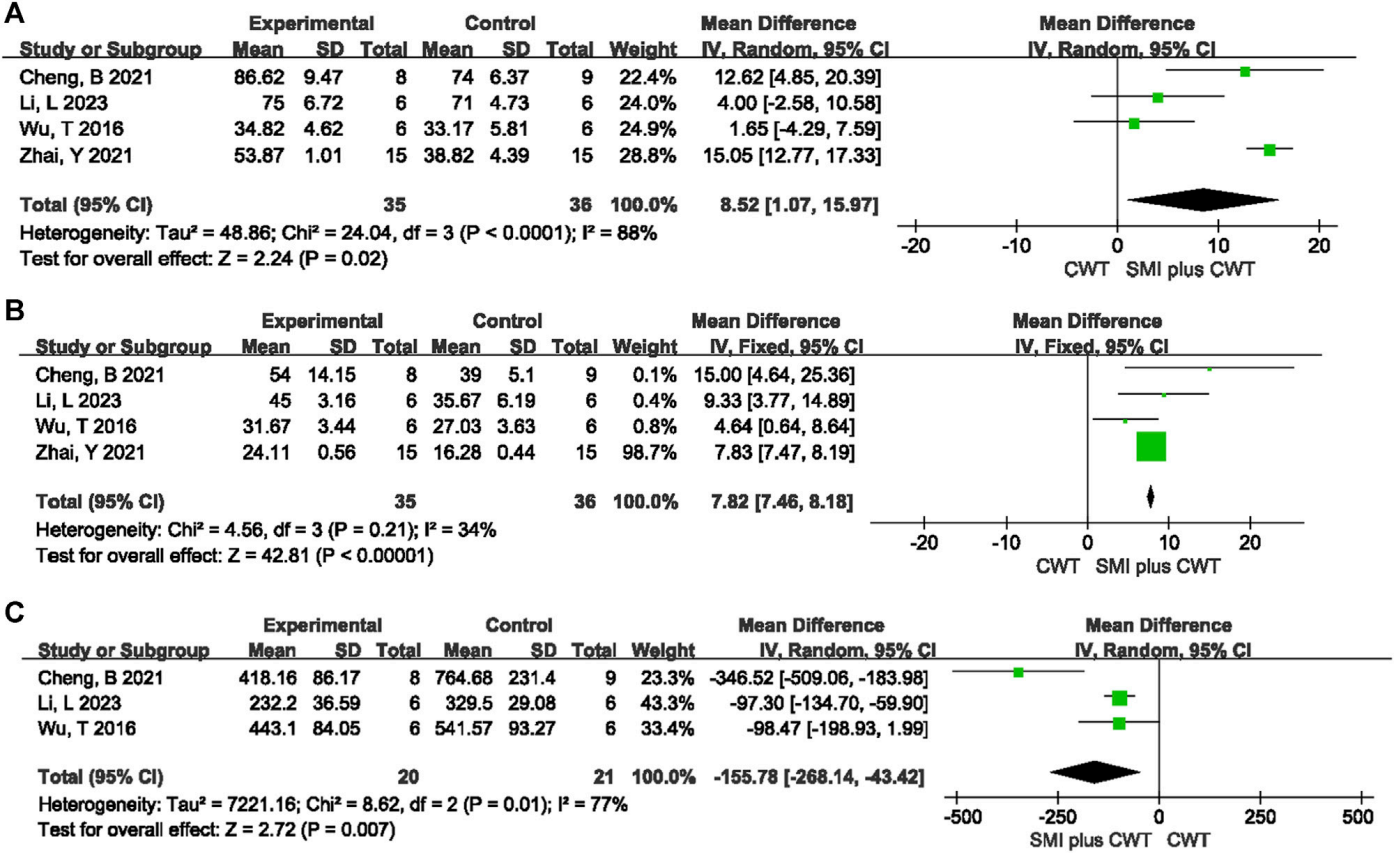
The meta-analysis result of cardiac function indicators and NT-proBNP in rats **(A)**: LVEF; **(B)**: LVFS; **(C)**: NT-proBNP.

Four animal studies with a total of 71 rats were included for LVEF and 4 animal studies with a total of 71 rats were included for LVFS. The results of the meta-analysis showed that the experimental groups had a greater increase in LVEF [MD = 8.52, 95% CI (1.07, 15.97), *p* = 0.02] and LVFS [MD = 7.82, 95% CI (7.46, 8.18), *p* < 0.00001] compared with the control groups (Figure 10).

##### 3.5.1.2 NT-proBNP

A total of 3 animal studies involving 41 rats were included. The results of the random-effects model meta-analysis showed that the experimental groups were able to reduce NT-proBNP better than the control groups [MD = −155.78, 95%CI (−268.14 −43.42), *p* = 0.007] (Figure 10).

#### 3.5.2 Mechanistic indicators in rats

**FIGURE 11:**
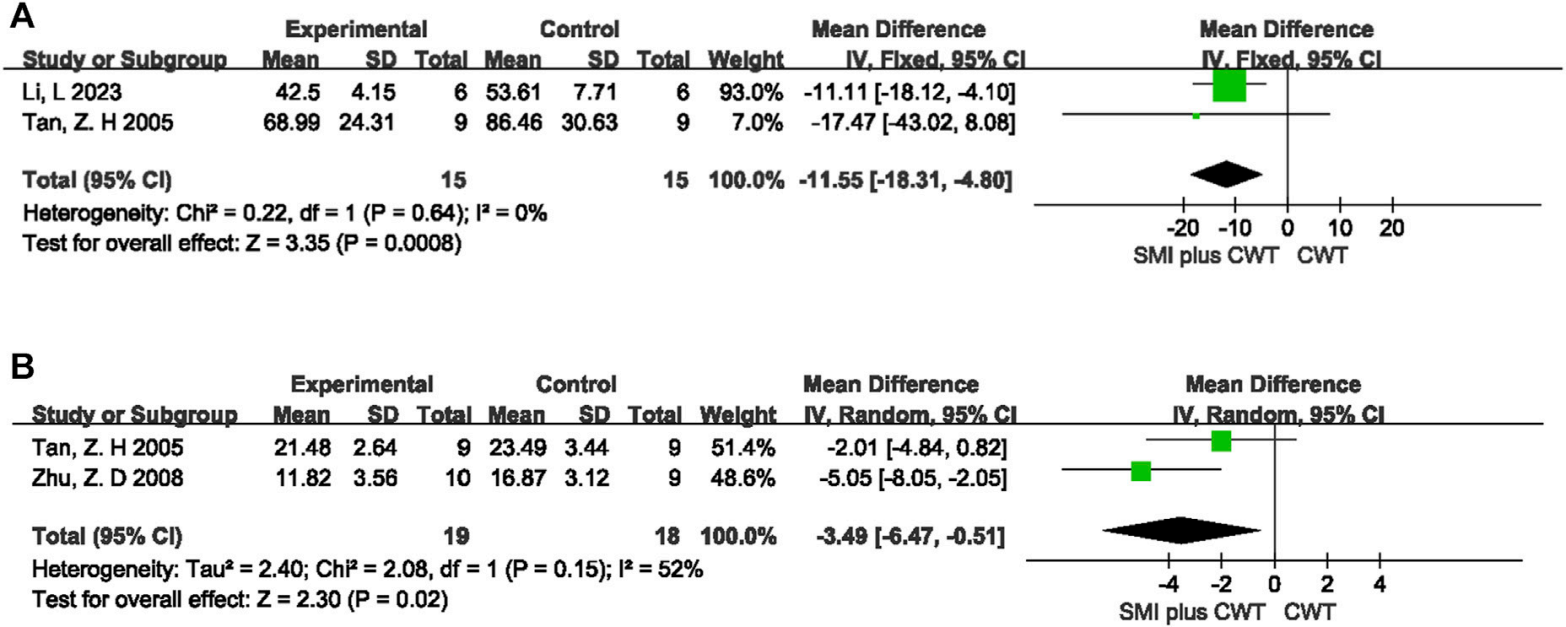
The meta-analysis result of mechanistic indicators in rats **(A)**:ET; **(B)**:TNF- α

ET was observed in a total of 2 animal studies involving 30 rats, while TNF-α was observed in 2 animal studies involving 37 rats. The results of the meta-analysis showed that the experimental groups had a better reduction in ET [MD = −11.55, 95%CI (−18.31 −4.80), *p* = 0.0008] and TNF-α [MD = −3.49, 95%CI (−6.47 −0.51), *p* = 0.02] compared with the control groups (Figure 11).

#### 3.5.3 Dog study results

In 3 animal studies regarding dogs, one study (Wang et al., 2012) found that SMI increased the concentration of endogenous digitalis-like substances in the myocardium of canine heart failure models. Furthermore, one study (Wang et al., 2010b) demonstrated that SMI could downregulate the levels of TNF-α, interleukin-1beta (IL-1β), and IL-6 in the blood of canine heart failure models, with certain dose-response and time-response relationships. Another study (Zhang et al., 2009) reported that treatment with different doses of SMI were all able to improve LVEF and atrial natriuretic peptide (ANP) when compared with the model group (*p* < 0.05), and it showed that efficacy improved in a dose-dependent manner.

In conclusion, SMI has the ability to improve cardiac function and biomarkers of heart failure in dog models of heart failure. The mechanism of action may involve increased endogenous digitalis-like substances in the myocardium of canine models and improved inflammation-related factors.

### 3.6 Subgroup analysis

**FIGURE 12:**
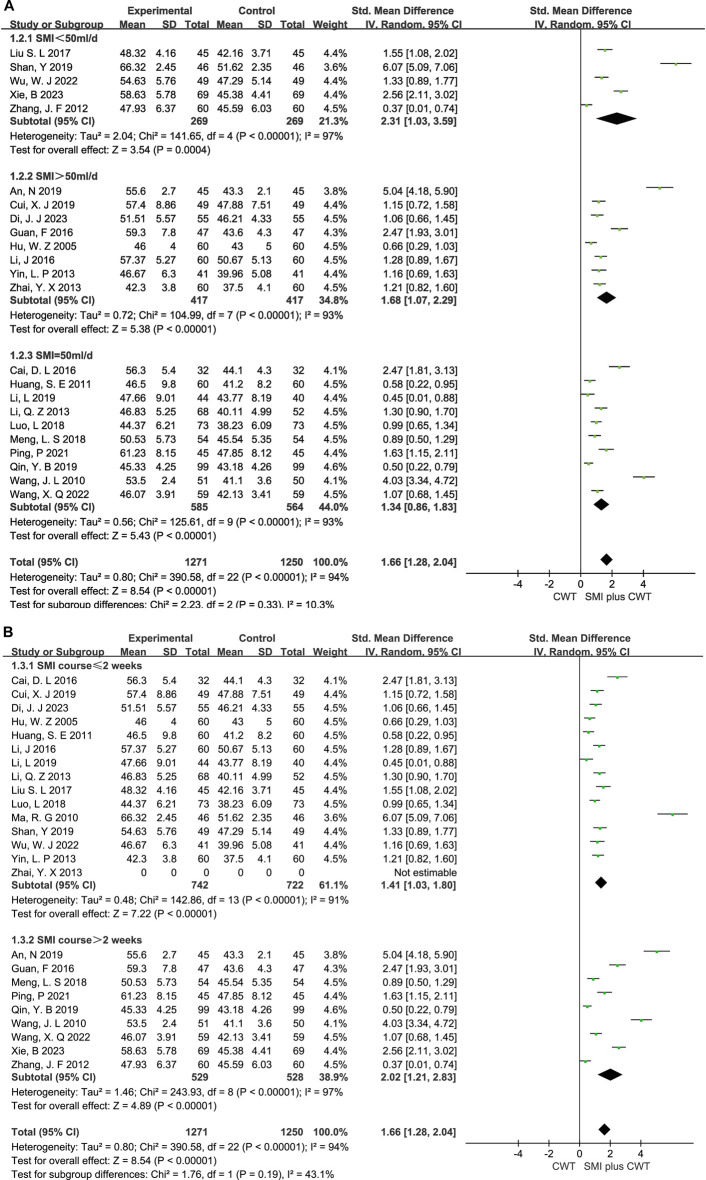
The meta-analysis result of the course and dose subgroups of LVEF **(A)**:Dose subgroups; **(B)**:Course subgroups.

In clinical trials, the I^*2*^ = 97% for LVEF. Therefore, subgroup analysis was performed based on the differences in treatment duration and dosage (Figure 12). In the subgroup analysis of treatment duration, it was further divided into two subgroups: one with a duration ≤2 weeks of treatment (*p* < 0.00001, I^2^ = 91%) and another one with a duration >2 weeks of treatment (*p* < 0.00001, I^2^ = 97%). The dose subgroup was further divided into three subgroups: SMI <50 mL/d (*p* < 0.00001, I^2^ = 97%), SMI >50 mL/d (*p* < 0.00001, I^2^ = 93%), and SMI = 50 mL/d (*p* < 0.00001, I^2^ = 93%).

### 3.7 Sensitivity analysis

To assess the impact of heterogeneity, outcome indicators with high heterogeneity (LVEF, BNP/NT-proBNP, LVEDD, LVESD, hs-CRP, TNF-α, IL-6) were systematically excluded from the included literature. However, the heterogeneity of these measures did not change.

### 3.8 Publication bias

**FIGURE 13:**
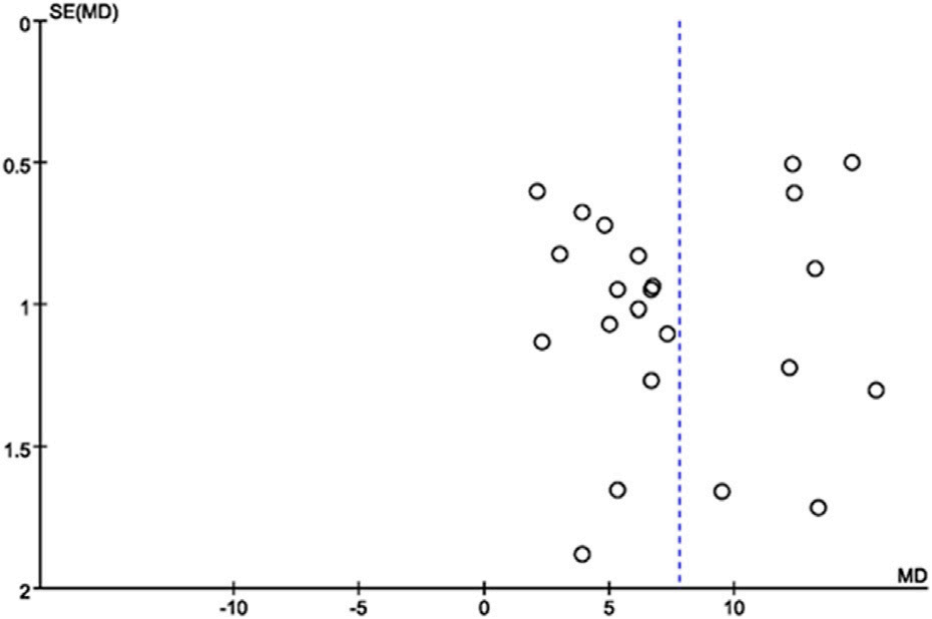
Funnel plot of the LVEF.

Publication bias of the included literature was assessed using a funnel plot. The funnel plot was constructed using LVEF from the 23 included studies as the outcome indicator. The results showed that the distribution of points in the funnel plot was not completely symmetric around the center line, indicating the presence of publication bias in the included literature (Figure 13).

## 4 Discussion

### 4.1 Results of the study

This study included a total of 25 clinical trials involving 2,705 patients and 11 animal studies which involved 201 animals. It systematically evaluated the efficacy and safety of SMI as an adjunctive therapy for CHF. The results of the clinical trials showed that SMI could improve cardiac function parameters such as LVEF, LVFS, LVEDV, LVESV, LVEDD and LVESD, reduce BNP/NT-proBNP levels and improve inflammatory markers such as hs-CRP, TNF-α, IL-6 and ET with statistically significant differences. In addition, SMI demonstrated a favorable safety profile. In animal studies, SMI demonstrated significant improvements in cardiac function parameters such as LVEF and LVFS, and the heart failure biomarker NT-proBNP, all of which were statistically significant compared to the control group.

### 4.2 Clinical effects of SMI and potential mechanisms of SMI in CHF

#### 4.2.1 Clinical effects of SMI

Shenmai Injection’s primary components are extracted from Ginseng and Ophiopogon japonicus. Ginseng is known for its ability to tonify vital energy and consolidate and generate body fluids, while Ophiopogon japonicus is known for nourishing Yin and generating fluids. Altogether, this combination results in synergistic effects that promote blood circulation, nourish Qi, and generate fluids. Shenmai Injection is indicated for the treatment of angina pectoris, myocardial infarction, viral myocarditis, and heart failure. Its mechanism of action involves enhancing cardiac contractility and blood pressure, improving coronary blood flow, increasing the body’s tolerance to hypoxia, and reducing myocardial oxygen consumption. At the same time, it has protective and reparative effects on myocardial cells and exhibits certain anti-arrhythmic properties.

**FIGURE 14:**
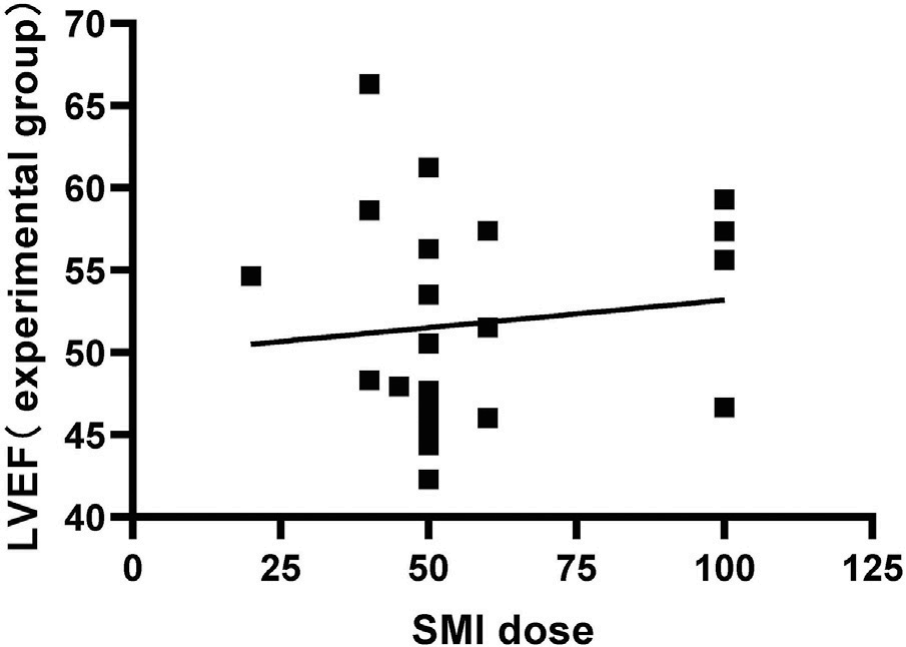
SMI Dose-LVEF relationship.

We plotted the relationship between the SMI dose reported and the LVEF of the test group in the included literature (Figure 14).

#### 4.2.2 Mechanistic studies of SMI

SMI is a traditional Chinese medicine composed of Ginseng and Ophiopogon. The main constituents of Ginseng include saponins, polysaccharides and essential oils. Among them, the active components closely associated with CHF are Ginsenoside Rg1(GRg1) and Ginsenoside Rb1(GRb1). The mechanism of GRg1 in improving CHF mainly involves improving left ventricular function (Zhang et al., 2013), inhibiting myocardial fibrosis (Zhang et al., 2013), promoting angiogenesis (Yin et al., 2011), and modulating the signaling pathways related to myocardial hypertrophy (Tang et al., 2016). GRg1 can suppress TNF-α mediated NF-κB activation, thereby attenuating cardiac hypertrophy induced by abdominal aortic constriction (Tang et al., 2016). On the other hand, GRb1 can improve energy metabolism in cardiac myocytes (Li et al., 2023b), inhibit inflammation (Ke et al., 2020; Wang et al., 2021), suppress myocardial hypertrophy (Wang et al., 2021), and inhibit myocardial fibrosis (Ke et al., 2020). Relevant studies suggest that GRb1 improves energy metabolism in cardiac myocytes by activating the PPARα pathway, possibly through the inhibition of Fas-associated death domain (Li et al., 2023b). GRb1 may alleviate age-related cardiac dysfunction by inhibiting fibrosis and inflammation, which may be related to the regulation of the NF-κB pathway (Ke et al., 2020).

Ophiopogon contains effective chemical components such as steroidal saponins, high isoflavones and carbohydrates (Chen et al., 2016). These components may improve CHF by reducing oxidative stress and inhibiting inflammatory responses. Steroidal saponins extract from Ophiopogon japonicus root shows significant protective effects against doxorubicin-induced chronic heart failure, possibly through inhibition of the p38 protein kinase (p38 MAPK) signaling pathway, reduction of oxidative stress, and anti-inflammatory responses (Wu et al., 2019). Furthermore, Ophiopogonin D can reduce doxorubicin-induced ROS production, activate c-jun N-terminal kinase (JNK) and ERK pathways, thereby significantly reducing potential damage of the mitochondrial membrane through antioxidant effects (Zhang et al., 2015).

#### 4.2.3 Potential mechanisms of SMI in CHF

##### 4.2.3.1 Anti-cell apoptosis

Cell apoptosis refers to the genetically controlled, autonomous, and orderly death of cells aiming to maintain internal environmental stability. Bcl-2 is closely related to cell apoptosis. One study found that SMI could exert anti-apoptotic effects by upregulating the Bcl-2/Bax ratio and upregulating caspase-3 protein expression (Chen et al., 2010). In addition, one study has shown that SMI can downregulate the expression of JNK protein and p38 MAPK, and modulate the JNK/p38 MAPK signaling pathway, thereby regulating the signaling pathway of myocardial cell apoptosis and ameliorating the symptoms of CHF in rats (Tan, 2005).

##### 4.2.3.2 Antioxidant effects

Some research results indicate that SMI can increase the levels of superoxide dismutase (SOD) and nitric oxide (NO) and reduce the levels of serum malondialdehyde (MDA) and ET (Li et al., 2015; Li et al., 2023a). SMI may have achieved this cardioprotective role by scavenging free radicals, reducing lipid peroxidation, and improving vascular endothelial dysfunction.

##### 4.2.3.3 Anti-inflammatory effects

Inflammatory responses play a critical role in the onset and development of CHF and are strongly associated with poor prognosis. Therefore, inhibition of inflammatory responses is of great importance to the prevention and treatment of CHF. Some research shows that SMI can inhibit the inflammatory response in animal CHF model, resulting in decreased levels of C-reactive protein (CRP), TNF-α, IL-6, and IL-1β in the serum and improved cardiac function (Zhu et al., 2008; Wang et al., 2010b; Zhai et al., 2021).

##### 4.2.3.4 Improving myocardial metabolism

**FIGURE 15:**
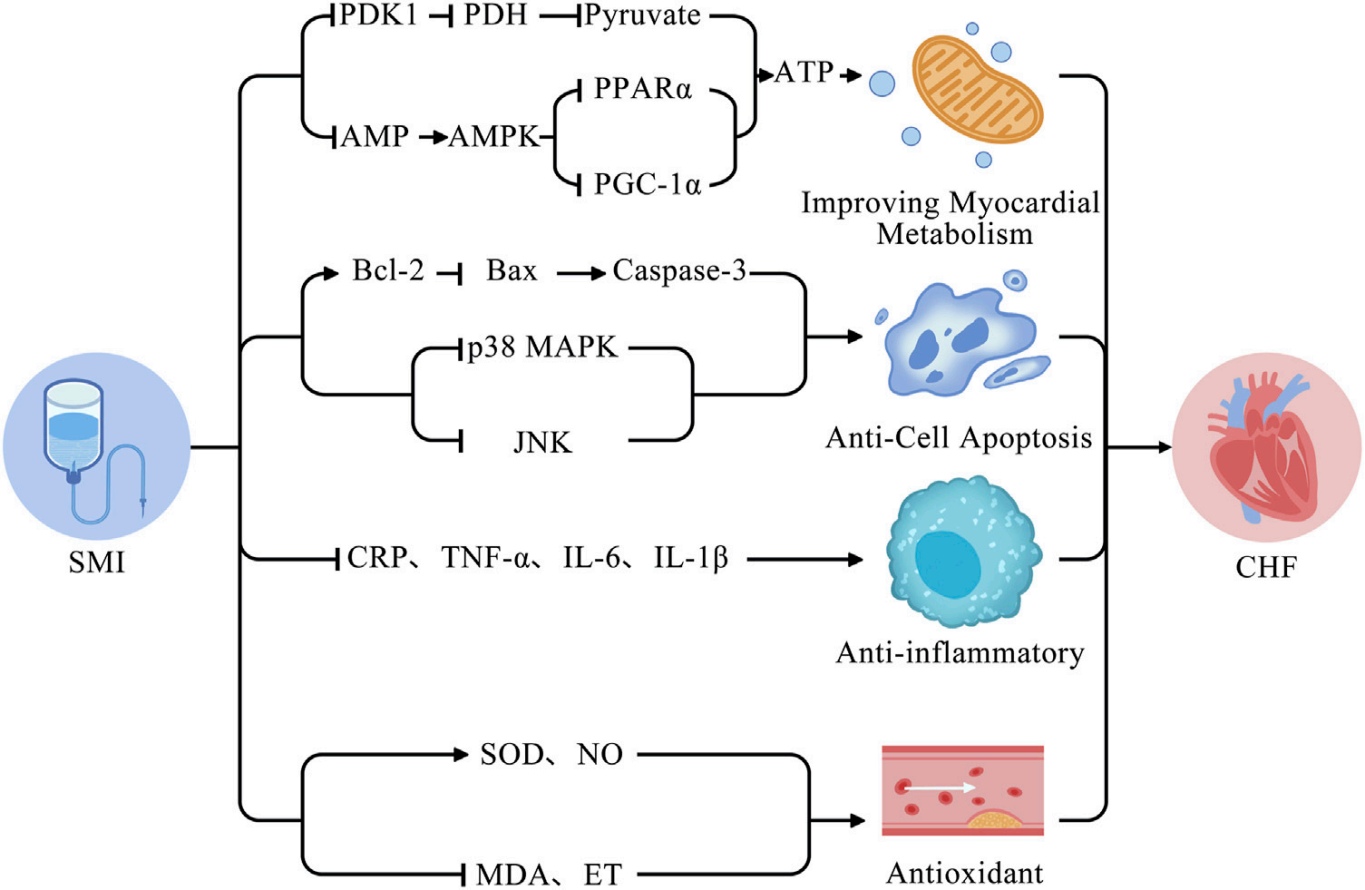
Potential mechanisms of SMI for the treatment of CHF.

Alterations in myocardial metabolism in CHF may exacerbate ventricular remodeling and progress heart failure. SMI exerts a multi-component, multi-target regional regulation of metabolism in CHF, increasing metabolic flexibility. One study showed that SMI restores mitochondrial structure and function, promotes ATP generation, and improves metabolism by downregulating the expression of adenosine monophosphate (AMP) and upregulating the expression of adenosine monophosphate-activated protein kinase (AMPK), thereby downregulating peroxisome proliferator-activated receptor alpha (PPARα) and proliferator-activated receptor gamma coactivator 1-alpha (PGC-1α) (Cheng et al., 2021). Similarly, a study showed that SMI effectively decreased pyruvate dehydrogenase kinase 1 (PDK1), thereby downregulating pyruvate dehydrogenase (PDH) in an oxidatively damaged H9c2 cardiomyocyte model (Zhao et al., 2018). The mechanism behind these effects may decrease pyruvate levels and increase ATP levels, thereby maintaining the stability of mitochondrial function and cellular metabolism. The following is a diagram of the potential mechanisms of SMI for the treatment of CHF (Figure 15).

### 4.3 Differences from previous studies

Previous studies mainly consisted of meta-analyses of clinical research (Chen et al., 2014), and several high-quality relevant RCTs have been published in recent years (Ma et al., 2010; Zhang et al., 2021). This study conducted a comprehensive search of currently published research both in Chinese and English, including preclinical animal studies (Tan, 2005; Zhu et al., 2008; Zhang et al., 2009; Wang et al., 2010b; Wang et al., 2012; Xu, 2015; Wu, 2016; Cheng et al., 2021; Zhai et al., 2021; Li et al., 2023a; Hu et al., 2023). This research represents a novel and comprehensive contribution to the field, and provides a thorough account of the efficacy, safety, and relevant mechanisms of SMI.

### 4.4 Clinical and research implications

#### 4.4.1 Implications of preclinical studies

##### 4.4.1.1 Preparation of animal models

This study discovered certain differences between the animal models of heart failure and clinical patients. CHF is a clinical syndrome that develops over time and is often associated with comorbidities such as diabetes and hyperlipidemia. Therefore, future animal studies in CHF should use models that more accurately reflect the pathological mechanisms and disease progression to better elucidate the pharmacological mechanisms of SMI.

##### 4.4.1.2 Quality and quantity of animal studies

The rationale and standardization of animal studies have a direct impact on the conversion from preclinical to clinical research. In this study, the evaluation of the included animal studies using the SYRCLE tool shows that most of the methodological quality of the animal studies is of moderate quality. Therefore, in future experimental studies, researchers should strictly adhere to the standards of animal studies when designing their study protocols. This includes emphasis on allocation concealment, random housing, blinding of investigators, and blinding of outcome assessors, as well as detailed documentation of relevant information in the manuscript.

The mechanism of SMI treatment for CHF is not yet conclusive, and further in-depth preclinical studies are needed to explore the signaling pathways associated with ventricular remodeling, identify the key bioactive compounds responsible for its effects, and elucidate the underlying mechanisms.

#### 4.4.2 Insights from clinical research

##### 4.4.2.1 Selection of outcome measures in Clinical studies

Although a large number of clinical studies have evaluated the efficacy and safety of SMI in the treatment of CHF, their outcome indicators focused primarily on cardiac function and biomarkers of heart failure, without considering indicators such as cardiovascular disease mortality or all-cause mortality. As a result, the impact of SMI on the prognosis of CHF patients cannot be proven. It is recommended that future clinical research pay more attention to clinical prognostic indicators to provide high-quality evidence for the clinical application of SMI.

##### 4.4.2.2 Administration and duration of SMI treatment for CHF

The pharmacological effects of Chinese herbs depend on the dosage and administration methods used in practice. In previous studies, there have been controversy regarding the dosage and administration of SMI due to the lack of comprehensive summary methods. This study shows that most studies have used SMI doses ranging from 20 to 100 mL diluted in 100–250 mL of 5% glucose solution, all of which have shown favorable results. Chinese herbal medicines typically contain complex compounds that exert therapeutic effects after absorption, distribution, metabolism, and excretion. Therefore, it takes some time for the therapeutic effects to manifest. In this study, the average duration of SMI treatment was found to be 3 weeks, suggesting that a 3-week course of SMI treatment may be beneficial for CHF therapy.

### 4.5 Limitations of the study

Firstly, the study may have overestimated the clinical efficacy of SMI due to publication bias in the literature. Secondly, caution should be exercised when interpreting the results of the included clinical studies due to small sample sizes, lack of sample size estimation, differences in preparation, and overall low quality of the included literature. Thirdly, the short follow-up period of the included studies did not allow for an assessment of the impact on long-term clinical prognosis such as the mortality in patients with CHF, among others. Therefore, future long-term follow-up studies should be conducted. Fourthly, only Chinese and English literature were included, while literature in other languages were excluded, which may lead to selection bias. Fifthly, the included studies span from 2005 to 2023, during which the treatment plans and medications for CHF may have changed over time. Sixthly, the quality assessment results of some of the included animal studies were moderate, and many of them neglected the importance of randomization and blinding. Finally, some of the included clinical trials lacked the differentiation of traditional Chinese medicine syndromes, making it difficult to provide tailored medication guidance for specific syndrome types.

## 5 Conclusion

This study is the first to include preclinical and clinical evidence of SMI in the treatment of CHF. The results suggest that SMI may ameliorate cardiac function, and in turn improve the quality of life for CHF patients. Preclinical studies suggest that the mechanisms underlying the beneficial effects of SMI on CHF may include anti-apoptotic effects, antioxidant effects, anti-inflammatory effects, and improvement of myocardial metabolism. Finally, our study summarizes the major limitations of existing research and provides suggestions for improving future studies.

## Data Availability

The original contributions presented in the study are included in the article/Supplementary Material, further inquiries can be directed to the corresponding author.
