# Supplementary material for: Effects of Shenmai injection against chronic heart failure: a meta-analysis and systematic review of preclinical and clinical studies
Source: Front Pharmacol. 2024 Feb 6;14:1338975. doi: 10.3389/fphar.2023.1338975 (PMC10880451; doi:10.3389/fphar.2023.1338975)
Supplement: Supplementary file 1 [file DataSheet7.PDF]

The search strategies of Shenmai injection for chronic heart disease

Databases	search strategies	Hit Counts
**Pubmed (https://pubmed.ncbi.nlm.nih.gov/advanced/)**	#1: All Fields= “Shenmai Injection” OR “shenmai Injection” OR “ShenMai Injection”<br>#2: All Fields= “Heart failure” OR “Cardiac failure”<br>#3: #1 and #2	27
**Web of science (https://www.webofscience.com/)**	#1: TS= “Shenmai Injection” OR “shenmai Injection” OR “ShenMai Injection”<br>#2: TS= “Heart failure” OR “Cardiac failure”<br>#3: #1 and #2	30
**Embase (https://www.embase.com/#advancedSearch)**	#1: Broad search= ‘Shenmai Injection’ OR ‘shenmai Injection’ OR ‘ShenMai Injection’<br>#2: Broad search= ‘Heart failure’ or ‘Cardiac failure’<br>#3: #1 and #2	55
**The Cochrane Library (https://www.cochranelibrary.com/advanced-search)**	#1: “Shenmai Injection” OR “shenmai Injection” OR “ShenMai Injection”<br>#2: “Heart failure” OR “Cardiac failure”<br>#3: #1 and #2	15
**CNKI (https://www.cnki.net/)**	(FT= ('参麦注射液') AND FT=('慢性心力衰竭' + '慢性心竭')) AND (SU=('参麦注射液') OR TKA=('慢性心力衰竭' + '慢性心竭'))	1478
**WanFang (https://w.wanfangdata.com.cn/)**	全部: ("参麦注射液") and 全部: ("慢性心衰" or "慢性心力衰竭")	271
**VIP (http://www.cqvip.com/)**	U=(参麦注射液) and U=(慢性心衰 OR 慢性心力衰竭)	417
**SinoMed (http://www.sinomed.ac.cn/cross/advancedSearch.jsp)**	"参麦注射液"[全部字段:智能] AND("慢性心衰"[全部字段:智能] OR "慢性心力衰竭"[全部字段:智能])	224
**the Chinese Clinical Trial Registry (https://www.chictr.org.cn/searchproj.html)**	在“干预措施”检索框中，输入“参麦注射液”。	12
**The World Health Organization International Clinical Trials Registry Platform (https://trialsearch.who.int/AdvSearch.)**	#1 (Title): Shenmai Injection OR shenmai Injection OR ShenMai Injection<br>#2 (Condition): Heart failure OR Cardiac failure<br>#3: #1 and #2	1

**ClinicalTrials.gov (https://trialsearch.who.int/AdvSearch.aspx)**	#1 (Intervention/treatment): Shenmai Injection or shenmai Injection or ShenMai Injection<br>#2 (Title/Acronym): Heart failure OR Cardiac failure<br>#3: #1 and #2	0
