# Supplementary material for: Effects of Shenmai injection against chronic heart failure: a meta-analysis and systematic review of preclinical and clinical studies
Source: Front Pharmacol. 2024 Feb 6;14:1338975. doi: 10.3389/fphar.2023.1338975 (PMC10880451; doi:10.3389/fphar.2023.1338975)
Supplement: Supplementary file 2 [file DataSheet2.PDF]

Table 2: Basic characteristics of 11 animal studies

Study ID	Model	Species（Sex, Weight, N=Experimental/Control group）	Anesthetic	Treatment group method	Control group method	Duration	Efficacy Indicators
Hu, S. Y 2023	A	Salt-sensitive rats (male,200-240g, n=6/6)	Urethane (20%, 6 mL/kg)	SMI (6mL/kg/d)	Sterile water (6mL/kg/d)	15 days	
Li, L 2023	A	Salt-sensitive rats (male,210-230g, n=6/6)	NM	SMI (6mL/kg/d)	Sterile water for injection (6mL/kg/d)	15 days	①②③④
Cheng, B 2021	A	Salt-sensitive rats (male,220-240g, n=8/9)	NM	SMI (6mL/kg/d)	Sterile water (6mL/kg/d)	15 days	①②③
Zhai, Y 2021	B	Wistar rats (male,220-250g, n=15/15)	Isoflurane	SMI (5.4 mL/kg/d)	0.9% sodium chloride solution (5.4 mL/kg/d)	2 weeks	①②
Wu, T 2016	B	Sprague-Dawley rats (male,200-220g, n=6/6)	Chloral hydrate (10%,0.35ml/100g)	SMI (6mL/kg/d)	Sterile water for injection (6mL/kg/d)	15 days	①②③
Xu, J. J 2015	A	Wistar rats (male,280-320g, n=8/8)	NM	SMI (2.4mL/300g/d)	0.9% sodium chloride solution (2.5 mL/d)	4 weeks	
Wang, X. L 2012	B	Crossbreed dog (male and female,11.1-12.3kg, n=3/3)	NM	SMI 1.511mL/kg	20 mL 0.9% NaCl	1 time	
Wang, H. H 2010	B	Crossbreed dog (male and female,10.98-12.62kg, n=15/5)	NM	SMI LDG/ MDG/ HDG(n=5/5/5):0.517/1.034/1.511mL/kg/d	20 mL 0.9% NaCl/d	1 week	④
Zhang, Z. P 2009	B	Crossbreed dog (male and female,11-12.6kg, n=15/5)	NM	SMI LDG/ MDG/ HDG(n=5/5/5):0.517/1.034/1.511mL/kg/d	20 mL 0.9% NaCl/d	1 week	①
Zhu, Z. D 2008	A	Wistar rats (male,180-220g, n=29/9)	Pentobarbital (1.5%, 35mg/kg)	SMI LDG/ MDG/ HDG(n=9/10/10):2/4/8 mL/kg/d	5% glucose solution/d （8mL/kg/d）	40 days	④
Tan, Z. H 2005	A	Wistar rats (male,180-250g, n=9/9)	Pentobarbital sodium(3%, 1mL/kg)	SMI (8mL/kg/d)	Distilled water 1.5ml/d	4 weeks	④

Note: SMI: Shenmai Injection; Model A: Pressure overload-induced heart failure model; Model B: Myocardial infarction-induced heart failure model; NM: no mention**;** LDG: Low-dose group; MDG: Middle-dose group; HDG: High-dose group; ①: LVEF; ②: LVFS; ③: NT-proBNP; ④: Mechanism indicators (TNF-α、ET).
