# Supplementary material for: Effects of Shenmai injection against chronic heart failure: a meta-analysis and systematic review of preclinical and clinical studies
Source: Front Pharmacol. 2024 Feb 6;14:1338975. doi: 10.3389/fphar.2023.1338975 (PMC10880451; doi:10.3389/fphar.2023.1338975)
Supplement: Supplementary file 3 [file DataSheet4.PDF]

Table 4: Methodological evaluation of included clinical studies (RoB-2 method)

Study ID	Randomization process	Assignment to intervention	Adhering to intervention	Missing outcome data	Measurement of outcome	Selection of the reported result	Overall score
Di, J. J 2023	Low risk	Some concerns	Low risk	Low risk	Low risk	Some concerns	Some concerns
Xie, B 2023	Some concerns	Some concerns	Low risk	Low risk	Some concerns	Some concerns	Some concerns
Wu, W. J 2022	Low risk	Some concerns	Low risk	Low risk	Some concerns	Some concerns	Some concerns
Wang, X. Q 2022	Low risk	Low risk	Low risk	Low risk	Low risk	Low risk	Low risk
Ping, P 2021	Some concerns	Some concerns	Low risk	Low risk	Some concerns	Some concerns	Some concerns
Li, L 2019	Some concerns	Some concerns	Low risk	Low risk	Low risk	Some concerns	Some concerns
Cui, X. J 2019	Low risk	Low risk	Low risk	Low risk	Low risk	Low risk	Low risk
Qin, Y. B 2019	Low risk	Some concerns	Low risk	Low risk	Some concerns	Some concerns	Some concerns
An, N 2019	Some concerns	Some concerns	Low risk	Low risk	Some concerns	Some concerns	Some concerns
Shan, Y 2019	Some concerns	Some concerns	Low risk	Low risk	Some concerns	Some concerns	Some concerns
Meng, L. S 2018	Low risk	Low risk	Low risk	Low risk	Low risk	Low risk	Low risk
Luo, L 2018	Low risk	Low risk	Low risk	Low risk	Low risk	Low risk	Low risk
Liu S. L 2017	Some concerns	Some concerns	Low risk	Low risk	Some concerns	Some concerns	Some concerns
Li, J 2016	Low risk	Low risk	Low risk	Low risk	Low risk	Low risk	Low risk
Guan, F 2016	Some concerns	Some concerns	Low risk	Low risk	Some concerns	Some concerns	Some concerns
Cai, D. L 2016	Some concerns	Some concerns	Low risk	Low risk	Some concerns	Some concerns	Some concerns
Li, Q. Z 2013	Some concerns	Some concerns	Low risk	Low risk	Some concerns	Some concerns	Some concerns
Zhai, Y. X 2013	Low risk	Low risk	Low risk	Low risk	Low risk	Low risk	Low risk
Yin, L. P 2013	Some concerns	Some concerns	Low risk	Low risk	Some concerns	Some concerns	Some concerns
Zhang, J. F 2012	Low risk	Low risk	Low risk	Low risk	Low risk	Low risk	Low risk
Huang, S. E 2011	Low risk	Some concerns	Low risk	Low risk	Some concerns	Some concerns	Some concerns
Wang, J. L 2010	Low risk	Some concerns	Low risk	Low risk	Some concerns	Some concerns	Some concerns
Hu, W. Z 2005	Some concerns	Some concerns	Low risk	Low risk	Low risk	Some concerns	Some concerns
Zhang, L 2021	Low risk	Low risk	Low risk	Low risk	Low risk	Low risk	Low risk
Ma, R. G 2010	Some concerns	Some concerns	Low risk	Low risk	Low risk	Some concerns	Some concerns
