# Supplementary material for: Effects of Shenmai injection against chronic heart failure: a meta-analysis and systematic review of preclinical and clinical studies
Source: Front Pharmacol. 2024 Feb 6;14:1338975. doi: 10.3389/fphar.2023.1338975 (PMC10880451; doi:10.3389/fphar.2023.1338975)
Supplement: Supplementary file 4 [file DataSheet6.PDF]

**Table 6: Meta-analysis of adverse effects of each drug**

| Types of adverse reactions | Adverse reactions in T/T | Adverse reactions in C /C | P |
|---|---|---|---|
| Gastrointestinal reactions | 3/227 | 6/221 | P=0.35 |
| Fatigue | 1/118 | 3/118 | P=0.38 |
| Dizziness and headache | 6/245 | 11/245 | P=0.24 |
| Rash or pruritus | 6/257 | 3/247 | P=0.43 |
| Abnormal liver and kidney function | 1/69 | 0/69 | P=0.50 |
| Hypotension | 6/128 | 5/128 | P=0.77 |
| Palpitations | 2/125 | 4/125 | P=0.45 |
| Arthralgia | 1/32 | 2/32 | P=0.56 |

Note:T: treatment group; C: control group.
