# Supplementary material for: Effects of Shenmai injection against chronic heart failure: a meta-analysis and systematic review of preclinical and clinical studies
Source: Front Pharmacol. 2024 Feb 6;14:1338975. doi: 10.3389/fphar.2023.1338975 (PMC10880451; doi:10.3389/fphar.2023.1338975)
Supplement: Supplementary file 5 [file DataSheet3.PDF]

Table 3: Table of mechanisms and targets studied in animals

Study ID	Experiment Content	Mechanism for SMI	Target for SMI
Li, L 2023	The experiment was conducted to create a pressure overload-induced heart failure model in rats, using SMI (n=6, 6 mL/kg/d) in the experimental group and sterilized water for injection (n=6, 6 mL/kg/d) in the control group, both with an intervention period of 15 days.	Antioxidant effect	ET*
Cheng, B 2021	This experiment created a pressure overload-induced heart failure model in rats, using SMI (n=8, 6 mL/kg/d) in the experimental group and sterilized water (n=9, 6 mL/kg/d) in the control group, both with an intervention period of 15 days.	Improvement of myocardial metabolism	AMPK**、PPARα**、PGC-1α**、AMP*、ATP* 、ADP
Zhai, Y 2021	This experiment was conducted to create a pressure overload-induced heart failure model in rats through isoflurane anesthesia, using SMI (n=15, 5.4 mL/kg/d) in the experimental group and sterilized water (n=15, 5.4 mL/kg/d) in the control group, both of which were intervened for 2 weeks.	Anti-inflammatory effects	CRP*、IL-6*
Wang, H. H 2010	This experiment was conducted to create a myocardial infarction-induced heart failure model in mongrel dogs, with low, medium, and high doses of SMI (n=5/5/5, 0.517/1.034/1.511 mL/kg) in the experimental group and 0.9% NaCl (n=5, 20 mL) in the control group, all for 1 week.	Anti-inflammatory effects	TNF-α*、IL-1β、IL-6*
Zhu, Z. D 2008	This experiment created a pressure overload-induced heart failure model in rats by pentobarbital anesthesia, with low, medium, and high dose of SMI (n=9/10/10), 2/4/8 mL/kg/d in the experimental group, and 5% glucose solution/d (n=9, 8 mL/kg/d) in the control group, both with an intervention period of 40 days.	Anti-inflammatory effects	TNF-α**
Tan, Z. H 2005	This experiment was conducted to create a pressure overload-induced heart failure model in rats by pentobarbital anesthesia, using SMI (n=9, 8 mL/kg/d) in the experimental group and distilled water (n=9, 1.5 mL/d) in the control group, both with an intervention period of 4 weeks.	Anti-inflammatory effects 、antiapoptotic effects	TNF-α* 、IL-6** 、JNK MAPK**、p38MAPK**、Bcl-2*、Bax*、Bcl-2/Bax**

Note：*: P＜0.05；**: P＜0.01. ADP: adenosine diphosphate; AMP: adenosine monophosphate; AMPK: adenosine monophosphate-activated protein kinase; ATP: adenosine triphosphate; Bcl-2: B lymphoblastoma-2; Bax: Bcl-2-associated X-protein; Bcl-2/Bax: B lymphoblastoma-2/Bcl-2-associated X-protein; CRP: C-reactive protein; ET: endothelin; IL-6: interleukin-6; IL-1β: interleukin-1β; JNK MAPK: Jun amino-terminal kinase mitogen-activated protein kinase; PPARα: peroxisome proliferator-activated receptor alpha; p38MAPK: p38 mitogen-activated protein kinase; PGC-1α: PPARγ coactivator 1α.
