# Supplementary material for: Effects of Shenmai injection against chronic heart failure: a meta-analysis and systematic review of preclinical and clinical studies
Source: Front Pharmacol. 2024 Feb 6;14:1338975. doi: 10.3389/fphar.2023.1338975 (PMC10880451; doi:10.3389/fphar.2023.1338975)
Supplement: Supplementary file 6 [file DataSheet1.PDF]

Table 1: Basic characteristics of 25 clinical studies

Study ID	Sample size (T/C)	Mean age(years)	Disease	T	C	Usage and dosage，duration	Outcomes
Di, J. J 2023	110（55/55）	T:60.9±8.1<br>C:61.7±8.8	CHF	SMI plus RT	RT	SMI 60 ml+5％GLU 125-200 ml, Qd, 2 weeks	①②⑤⑥
Xie, B 2023	138（69/69）	T:72.75±5.76<br>C:72.11±5.75	CHF	SMI plus RT	RT	SMI 40 ml+5％GLU 250 ml, Qd, 4 weeks	①③⑤⑥⑦
Wu, W. J 2022	98（49/49）	T:68.46±2.84<br>C:68.91±2.96	CHF	SMI plus RT	RT	SMI 20 ml+5％GLU 250 ml, Qd, 2 weeks	①②③⑤⑦
Wang, X. Q 2022	118（59/59）	T:68.42 ± 4.77<br>C:67.98 ±4.87	CHF	SMI plus RT	RT	SMI 50 ml+5％GLU 250 ml, Qd, 4 weeks	①②⑤
Ping, P 2021	90（45/45）	T:53.12±3.99<br>C:52.23±4.12	CHF	SMI plus RT	RT	SMI 50ml+0.9%NaCl 250ml, Qd, 4 weeks	①③⑤
Li, L 2019	84（44/40）	T:68.55±8.12<br>C:68.27±9.27	CHF	SMI plus RT	RT	SMI 50ml+5％GLU 100ml, Qd, 2 weeks	①②④⑤⑥⑦
Cui, X. J 2019	98（49/49）	T:59.98±9.11<br>C:61.02±7.69	CHF	SMI plus RT	RT	SMI 60ml+5％GLU 250ml, Qd, 2 weeks	①②③⑤⑦
Qin, Y. B 2019	198（99/99）	T:51.6±9.3<br>C:51.1±8.8	CHF	SMI plus RT	RT	SMI 50ml+5％GLU 250ml, Qd, 4 weeks	①②③⑤
An, N 2019	90（45/45）	T:60.12±2.50<br>C:59.97±2.3	CHF	SMI plus RT	RT	SMI 100ml+5％GLU 250ml, Bid, 4 weeks	①②③④⑤⑥
Shan, Y 2019	92（46/46）	T:71.35±5.24<br>C:71.46±5.17	CHF	SMI plus RT	RT	SMI 40ml+5％GLU 250ml, Qd, 2 weeks	①②③⑤⑦
Meng, L. S 2018	108（54/54）	T/C 57.70±8.90	CHF	SMI plus RT	RT	SMI 50ml+5％GLU 500ml, Qd, 4 weeks	①②⑤⑥
Luo, L 2018	146（73/73）	T:64.25±7.47<br>C:63.88±7.59	CHF	SMI plus RT	RT	SMI 50ml+5％GLU 250ml, Qd, 2 weeks	①②③⑦
Liu S. L 2017	90（45/45）	T:67.87±10.42<br>C:67.20±10.80	CHF	SMI plus RT	RT	SMI 40ml+5％GLU, Qd, 2 weeks	①②③④
Li, J 2016	120（60/60）	T:61.32±8.61<br>C:59.32±8.35	CHF	SMI plus RT	RT	SMI 100ml, Qd, 2 weeks	①②⑥
Guan, F 2016	94（47/47）	T:55.30±6.30<br>C:54.40±6.50	CHF	SMI plus RT	RT	SMI 100ml, Qd, 30 days	①②③⑦
Cai, D. L 2016	64（32/32）	T:65.2±7.4<br>C:65.1±7.5	CHF	SMI plus RT	RT	SMI 50ml+5％GLU 250 ml, Qd, 2 weeks	①②③⑤⑥⑦
Li, Q. Z 2013	120（68/52）	T:63.20±11.30<br>C:62.10±10.20	CHF	SMI plus RT	RT	SMI 50ml, Qd, 2 weeks	①②③
Zhai, Y. X 2013	120（60/60）	T:62.50±6.80<br>C:61.80±6.30	CHF	SMI plus RT	RT	SMI 40-60ml+5％GLU 250-500ml, Qd, 2 weeks	①②⑤
Yin, L. P 2013	82（41/41）	T:63.40±6.90<br>C:64.50±7.10	CHF	SMI plus RT	RT	SMI 100ml, Qd, 2 weeks	①②③⑥
Zhang, J. F 2012	120（60/60）	T:71.38±8.54<br>C:72.19±9.32	CHF	SMI plus RT	RT	SMI 45ml, 12 weeks	①②③⑤⑥
Huang, S. E 2011	120（60/60）	T:67.36±4.07	CHF	SMI plus RT	RT	SMI 50ml, Qd, 2 weeks	①②③

		C:69.27±3.96					
Wang, J. L 2010	101（51/50）	T:66.30±2.20<br>C:65.70±2.30	CHF	SMI plus RT	RT	SMI 50ml+5％GLU/0.9%NaCl 250ml, Qd, 15 days	①②③⑦
Hu, W. Z 2005	120（60/60）	T:65.28±17.5<br>C:63.13±16.40	CHF	SMI plus RT	RT	SMI 60ml+5％GLU 250ml, Qd, 2 weeks	①②⑤⑥
Zhang, L 2021	120（63/57）	T:64.73±8.99<br>C:64.82±9.77	CHF	SMI plus RT	RT	SMI 50ml, Qd, 2 weeks	③⑦
Ma, R. G 2010	64（33/31）	T:54.30±14.60<br>C:55.80±13.50	CHF	SMI plus RT	RT	SMI 30ml+5％GLU 250ml, Qd, 2 weeks	⑦

Note: T: treatment group; C: control group; SMI: Shenmai Injection; CHF: chronic heart failure; RT: routine treatment; GLU：glucose solution；NaCl：sodium chloride solution。①: LVEF; ②: BNP/NT-proBNP; ③: overall response rate; ④: 6-MWD; ⑤: Cardiac function; ⑥: Mechanism indicators (hs-CRP, TNF-α, IL-6, ET); ⑦: Adverse drug reactions。
