# Supplementary material for: Effects of Shenmai injection against chronic heart failure: a meta-analysis and systematic review of preclinical and clinical studies
Source: Front Pharmacol. 2024 Feb 6;14:1338975. doi: 10.3389/fphar.2023.1338975 (PMC10880451; doi:10.3389/fphar.2023.1338975)
Supplement: Supplementary file 7 [file DataSheet5.PDF]

Table 5: Methodological evaluations for inclusion in animal studies (SYRCLE tool)

Study ID	A	B	C	D	E	F	G	H	I	J	Overall score
Hu, S. Y 2023	1	1	0	0	0	1	0	1	0	1	5
Li, L 2023	1	1	0	0	0	0	0	1	0	1	4
Cheng, B 2021	1	0	0	0	0	0	0	1	0	1	3
Zhai, Y 2021	0	1	0	0	0	1	0	1	0	1	4
Wu, T 2016	1	1	0	0	0	1	0	1	0	1	5
Xu, J. J 2015	0	1	0	0	0	1	0	1	0	1	4
Wang, X. L 2012	0	1	0	0	0	0	0	1	0	1	3
Wang, H. H 2012	0	1	0	0	0	0	0	1	0	1	3
Zhang, Z. P 2009	0	1	0	0	0	0	0	1	0	1	3
Zhu, Z. D 2008	0	1	0	0	0	0	0	1	0	1	3
Tan, Z. H 2005	0	1	0	0	0	0	0	1	0	1	3

Note: A: sequence generation; B: baseline characteristics; C: allocation concealment; D: random housing; E: blinding of investigators; F: random outcome; G: blinding of outcome assessors; H: incomplete outcome data; I: selective outcome reporting; J: other sources of bias.
